# The Combination of Solid-State Chemistry and Medicinal Chemistry as the Basis for the Synthesis of Theranostics Platforms

**DOI:** 10.3390/biom11101544

**Published:** 2021-10-19

**Authors:** Dmitry Korolev, Viktor Postnov, Ilia Aleksandrov, Igor Murin

**Affiliations:** 1 Almazov National Medical Research Centre, Institute of Experimental Medicine, 2 Akkuratova Str., 197341 Saint Petersburg, Russia; korolev_dv@almazovcentre.ru (D.K.); postnovvn@mail.ru (V.P.); 2 Institute of Chemistry, Saint Petersburg State University, 7/9 Universitetskaya Emb., 199034 Saint Petersburg, Russia; igormurin@mail.ru

**Keywords:** therapy, early diagnosis, theranostics, targeted delivery, medicinal chemistry

## Abstract

This review presents the main patterns of synthesis for theranostics platforms. We examine various approaches to the interpretation of theranostics, statistics of publications drawn from the PubMed database, and the solid-state and medicinal chemistry methods used for the formation of nanotheranostic objects. We highlight and analyze chemical methods for the modification of nanoparticles, synthesis of spacers with functional end-groups, and the immobilization of medicinal substances and fluorophores. An overview of the modern solutions applied in various fields of medicine is provided, along with an outline of specific examples and an analysis of modern trends and development areas of theranostics as a part of personalized medicine.

## 1. Introduction

One of the most relevant trends in the development of modern medical chemistry is theranostics: an approach to drug development in which the created compositions have the potential to provide a joint solution to the problems of early diagnosis and targeted therapy for certain diseases. The concept of theranostics has paved the way for the development of multifunctional modified nanoparticles that combine diagnostic and therapeutic properties within a single material with a predefined set of properties. These serve as a basis for designing pharmacological compounds with predetermined effects on the organism. Modern medical practice includes the successful use of nanoparticles with various natures as carriers of drugs and fluorescent dyes. The contemporary level of solid-state chemistry makes it possible to design functional medical nanosystems; however, the search for best practices in synthesizing nanoparticles with the aim of having tailored properties, in accordance with present-day tasks, continues. In particular, these efforts are focused to a large extent on the possibility of using nanomaterials for specific drug delivery directly to the damaged area that requires pharmacological intervention.

## 2. Approaches to Theranostics, Current Trends, and Tools

**Figure 1:**
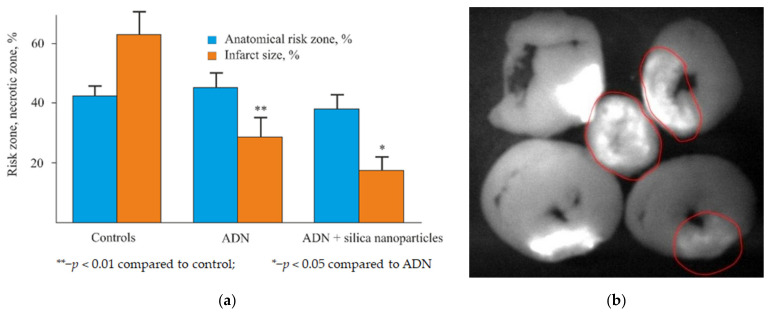
Example of theranostics in cardiology: (**a**) therapy for reduction of the infarction zone and (**b**) diagnostics for visualization of the infarction zone, using a fluorophore.

The scientific community has identified theranostics as a part of personalized medicine; theranostics refers to precision medicine in which drugs are selected individually for each patient, according to the predicted response thereof or to the risk of a disease [1,2]. The essence of the phenomenon does not really change, and theranostics, as an original trend, is a combination of early diagnosis and therapy. One example of such an interaction of areas in cardiology is a reduction of the infarction zone and its visualization (see Figure 1) [3,4].

Some modern publications have discussed ways to introduce the special term “theranostics”. In fact, the issue is rather of epistemic nature and regards the interface of chemistry and medicine [5], as the authors often use the term “paradigm”. This article presents a detailed analysis of publications in the field of theranostics, with the premise that theranostic interventions will allow for simultaneous diagnosis and treatment of diseases. It has been noted that, at present, many treatment methods are essentially theranostics, in the sense that the treatment process proceeds under control, and the effectiveness of treatment should lead to a deeper understanding of the patient’s condition. It can be concluded that such methods have long been known and are currently available in practice, as, in order to treat a disease, one must first diagnose it; therefore, the term “theranostics” is, in fact, unnecessary.

**Figure 2:**
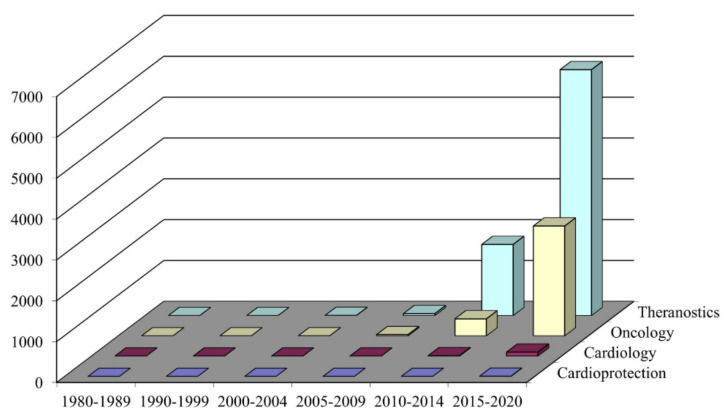
Dynamics of publications, using the PubMed database from 1980 to 2020.

Most studies that have focused on the effectiveness of targeted drug delivery based on nanoparticles are concentrated in the field of oncology. The problem of efficient targeted delivery of cardioprotectors to the myocardial ischemia–reperfusion area remains open. The PubMed database presented 6019 publications on theranostics, of which 2689 refer to the treatment for cancer, 99 are in the field of cardiology, and only six articles are related to cardioprotection (Figure 2).

In the general case, the development of modern platforms for theranostics is, of course, conducted in terms of chemistry. At least two of its branches are involved: The first branch is solid-state chemistry, as the issues in question are usually nanoparticles and solid-phase synthesis. The second branch of chemistry used for theranostics is medical or medicinal chemistry, as we are actually developing new medicines by using an already-known molecule or a pharmacophore fragment.

**Figure 3:**
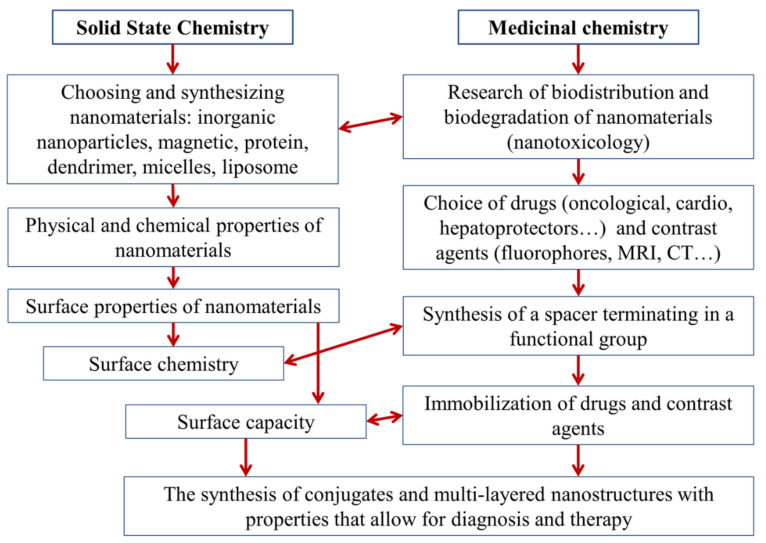
General synthesis diagram of platforms for theranostics.

A general theranostic platform synthesis scheme is illustrated in Figure 3. First, a nanotransporter is selected, and various nanomaterials have been used as the basis for theranostics systems. The variety of such materials is illustrated in Table 1. First, the biodistribution of the base in a living organism and its biodegradation or bio-elimination ability are studied. Then, chemical and physical properties of the nanomaterial surface are studied, and surface chemistry methods are selected for the immobilization of active substances and fluorophores. The latter are fixed on the surface of nanoparticles, using a spacer with a terminal functional group. This procedure is preceded by the screening and selection of drugs and fluorophores. The amount of immobilized substances determines the surface capacity and is characterized by such properties as the grafting density.

**Figure 4:**
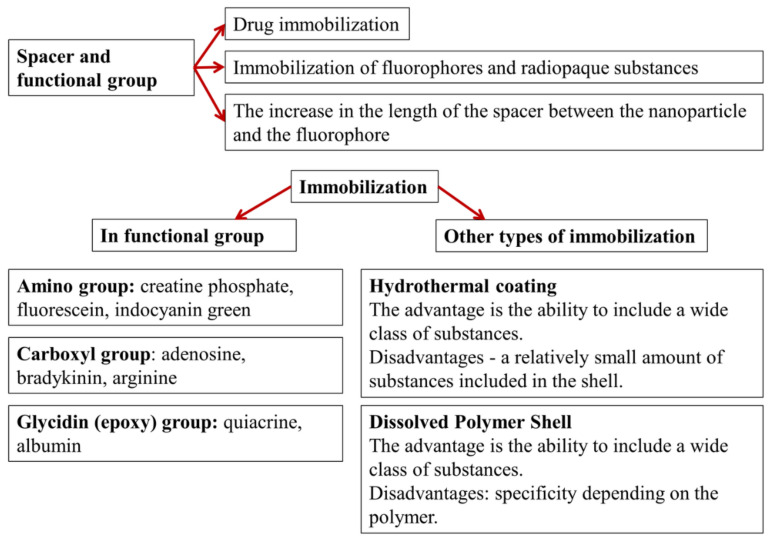
Purpose and types of spacers and shells with functional groups.

In general terms, the roles of spacer and immobilization methods are explained in Figure 4. Spacers with terminal functional groups are designed to immobilize active substances and various contrast agents (e.g., fluorescent, magnetic, X-ray, or ultrasound) are used. A separate important task is to extend the spacer, thus ensuring that distance is maintained between the carrier particle and the contrast agent, in order to avoid contrast damping. The immobilization of active substances and contrasts on the surface of nanocarriers can be achieved by using the functional group or by other methods. The most frequently used functional groups are amino, carboxyl, and (less commonly) glycidine groups [6,7]. Albumin is sometimes used as a spacer, acting as a transport protein with many functional groups [8]. Frequently used coatings include hydrothermal coatings and coatings from dissolved polymer shells, such as polylactic acid [9,10].

## 3. The Use of Silica in Medical Practice as a Basis for Theranostics

**Figure 5:**
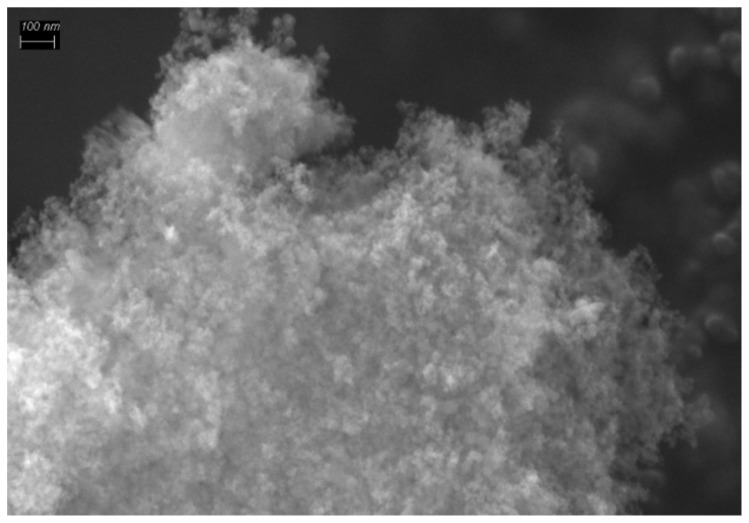
Scanning electron micrograph (SEM) of Aerosil.

At present, highly dispersed silica (HDS) Aerosil is widely used in biomedicine as a platform for theranostics and targeted drug delivery. It possesses a number of valuable properties, including biodegradability, a high specific surface (which determines a high capacity for a drug), and surface chemistry that allows for modification with various targeting ligands and fluorophores. The undoubted advantages of silica are its availability, low cost, and many years of application in medicine. We provide a detailed description of the development of systems for theranostics, using the example of nanosized silica. Aerosil 300 (Polysorb) has received widespread attention. This silica has a specific surface area of about 300 m^2^/g and consists of globular nanoparticles with a size of 8–10 nm that can form larger aggregates (Figure 5).

The surface of Aerosil contains Si–OH silanol groups, the concentration of which increases when in contact with water. These groups ensure the variety of interactions between finely divided silica (HDS) and biological objects, as well as its use in medicine. Research has shown that HDS is not genotoxic, and is practically non-toxic to the oral cavity, skin, and eyes [38]. Neither inhalation nor oral administration cause neoplasms (tumors). HDS is not mutagenic, and has shown promise as a basis for theranostic drug development. Moreover, also worthy of note are the properties that allow for the use of HDS in this area, including its biological compatibility, biodegradability in living organisms, option for varying the particle size, and large specific surface area (making it possible to obtain preparations with high content of biologically active substances), as well as the presence of functional groups necessary to create active centers designed for the immobilization of drugs and marker compounds.

To assess the prospects for using HDS in theranostics, we identify medical practice areas where it has already found its application below.

HDS has been most widely used as an enterosorbent, and it actively interacts with enteropathogenic organisms, such as *Escherichia coli*, *Staphylococcus aureus*, *Vulgar protea*, and *Pseudomonas aeruginosa*, forming aggregates of sorbent particles containing microbial cells [39]. The formation of agglutinates of microbial cells and SiO_2_ particles is accompanied by a sufficiently strong binding of micro-organisms. A study of the effect produced by finely divided silica on the wound microflora has shown that it equally intensively binds both Gram-positive cocci (Staphylococci) and Gram-negative bacilli (*Pseudomonas aeruginosa*). The drug not only binds microbes, but also adsorbs growth and reproduction factors thereof, as well as microbial exotoxins. Thus, highly dispersed silica, while not showing a direct antibacterial effect, significantly limits the manifestation of pathogenic properties of micro-organisms; this effect underlies the therapeutic effect of the drug when treating suppurative wounds and acute intestinal infections. It is well-known that many enterosorbents significantly alter the state of the digestive tract. For example, bran, pectin, guar gum chime, and other substances can change the state of the chyme, the speed of its passage, and (in some cases) the pH of the intestinal lumen [40,41]. Sorbents can also affect the state of the intestinal wall. With the prolonged intake of cellulose, the length of the small intestine decreases [42,43]. It has been found that dietary fiber can stimulate the growth of intestinal epithelial cells, thus increasing the mucosal surface area, or exert an “abrasive” effect [44]. Most enterosorbents stimulate intestinal motility, but some of them slow down the food transit time, causing stool delay (polyphepan) or constipation (coal sorbents) in patients [45,46]. Guar fibers in a therapeutic dose inhibit the secretion of pancreatic glucagon and increase calcium absorption [47,48]. Cellulose increases the activity of disaccharidases, whereas saponins inhibit lactase activity but increase alpha-amylase activity [49]. Chitin sorbents can modulate the activity of a wide range of enzymes—lipase, amylase, glucokinase, and prostaglandin synthetase [50]. Enterosorbents also affect the intestinal microflora by regulating its growth, both due to the sorption of micro-organisms and by changing their environment. Enterosorption may impair the absorption of trace elements and vitamins: the impact of this effect is determined by the type of sorbent, intake duration, and dose [45,51]. Thus, enterosorbents can have both positive and negative effects on an organism. Following the requirements of newly developed drugs, the toxicity of HDS has been studied in diverse species of animals (e.g., rats, pigs, and rabbits) in acute and chronic experiments. The results of a comprehensive study have shown that the therapeutic dose of this drug, equal to 100 mg/kg (and even exceeding that by 3–10 times), does not exert a toxic effect [39].

High sorption activity determines the possibility of using HDS in surgical practices. HDS significantly outperforms other sorbents in its ability to sorb protein; it exceeds debrisan by 4.5–5 times, and gelivin and celesorb by more than 10 times. Various blood serum proteins (e.g., albumin and globulins)—and, consequently, wound exudate proteins—are absorbed by HDS equally actively, which means that this substance is not selective by nature [39]. In clinical practice, HDS is used in combination with a gauze dressing for treating purulent wounds. A particularly important element of wound preservation is a multifunctional dressing. Thus, HDS most fully meets the requirements of wound preservation. HDS used as a primary dressing component binds a significant number of micro-organisms and prevents the invasion thereof into deeper tissue. It should be noted that the sorbent does not exhibit selective action against aerobic and anaerobic microflora. High and rapid water absorption by the sorbent promotes dehydration and mummification of non-viable tissues, conversion of wet necrosis into dry necrosis, dehydration of the edematous tissue, and relief of edema. Based on this, a dressing has been developed containing HDS mechanically connected to the gauze base [52]. It is applied over the wound surface and fixed with a bandage. This dressing can be applied at both pre-medical and first-medical aid stages.

The sorption properties of HDS and its ability to form a gel upon contact with water make its use in dentistry possible. HDS, in combination with a solution of chlorhexidine and a solution of decamethoxin, turned out to be a highly effective means for the treatment of herpetic stomatitis in children, erythema exsudativum multiforme, and an erosive-ulcerative form of lichen planus [39].

As is well-known, dental caries and their complications are currently treated with antibiotics, sulfonamides, enzymes, harmonious preparations, antiseptics, and herbal remedies. Different dosage forms, including solutions, pastes, emulsions, and ointments, administered by methods of surface application, irrigation, and inhalations for the treatment of dental diseases, and acute and chronic inflammatory processes of periodontal tissues and oral mucosa, do not allow for the fully realized pharmacological potential of these substances. This results from the fact that saliva constantly reduces the concentration of the substances, thus diminishing their effectiveness [53]. Therefore, there is a need to use drugs with pronounced detoxification properties and prolonged duration of action—these are exactly the properties of drugs immobilized on HDS. The use of HDS for drug immobilization provides a solution to the problem of uniform distribution of small amounts of biologically active substances in the system, and allows for extension of their duration of action [54,55,56]. Several biologically active drugs immobilized on HDS and intended for the treatment of acute deep caries and periodontal tissue diseases have been clinically tested. An efficient paste was developed that consists of a sorbent that carries biologically active trace elements (e.g., fluorine, copper, zinc, and manganese), antibiotics (a mixture of penicillin, streptomycin, and laevomycetin), and distilled water. This paste possesses high antimicrobial activity against dentin microflora in caries cavities, and the components display synergistic action. The clinical effectiveness of deep-tooth-decay treatment with the developed paste was evaluated after 24 months in 325 patients, where positive results were achieved in 89% of patients.

A large proportion of periodontal diseases involve periodontitis or inflammation of the periodontal tissues. HDS-immobilized preparations of plant and synthetic origin have been used for the treatment of periodontitis: eucalyptus, furazolidone, rivanol, lincomycin, etonium with urea, tincture of calendula and calamus, and salvin. The listed drugs serve as the constituents of suspensions and pastes for application to the gum and insertion into the gingival pockets. A total of 1085 patients with mild and moderate periodontitis aged 18–65 years were considered in the study. These patients were treated with both non-immobilized (first group, 285 people) and immobilized (second group, 800 people) drugs. The most pronounced and persistent therapeutic effect was obtained in patients who were treated with HDS-immobilized drugs. This was apparently due to prolongation of the main therapeutic properties of the drugs and the sorption of toxic products on HDS. The results showed that the treatment reduced inflammation in the gums and, in all cases, the effect of the immobilized drug exceeded that of the parent drug. The increase in therapeutic activity of the studied drugs, when immobilized on a sorbent, was probably due to not only by prolongation of their pharmacological action, but also by the HDS sorption properties. These properties allow for the removal of toxins of micro-organisms and tissue-decay products from the abnormal focus. Thus, it was found that the tested immobilized drugs of synthetic and herbal origin exert therapeutic effects for the treatment of teeth diseases, periodontal tissues, and oral mucosa that made it possible to recommend them for a wider dental practice [39].

The abovementioned HDS medical-application areas give us reason to believe that this nanomaterial can also be used as a carrier for targeted delivery of drugs used in theranostics. Obviously, in this case, it is necessary to modify the silica surface with functional groups capable of serving as immobilization centers of biologically active and marker compounds. Let us consider several studies in this area.

## 4. The Basics of Chemical Synthesis of Theranostics Platforms

A substance that can be used as an initial matrix in theranostics platforms is Aerosil A-380. Preliminary experiments have shown that Aerosil of this brand ensures a higher concentration of engrafted groups and immobilized drugs, as compared with Aerosils A-300 and A-175. The silica modification technique, based on a chemical assembly method, consists of several stages: chemisorption of (3-Aminopropyl)triethoxysilane, hydrolysis of unreacted alkoxy groups, addition of 3-(Boc-amino)octanoic acid as a spacer, deprotection, and deprotonation.

Chemosorption of (3-Aminopropyl)triethoxysilane is carried out in a flow-type reactor with a stationary carrier layer in dry nitrogen, at a temperature of 220 °C [57].

Fluorophores indocyanin green and fluorescein, as well as the anticancer drug Zn-protoporphyrin, can be immobilized on the surface of aminated Aerosil [57,58].

**Figure 6:**
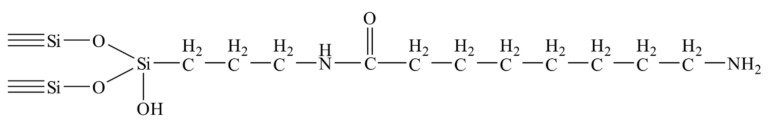
Aerosil with an engrafted spacer.

Spacers are prepared by using the methods of solid-phase peptide synthesis on silica matrices. Peptide bond formation is carried out by the symmetric anhydride method. After immobilization of 3-(Boc-amino)octanoic acid, the content of functional groups, determined by the analysis with the use of acid dye bright orange G, was equal to 0.053 mmol/g. Taking into account the content of amino groups after chemisorption of (3-Aminopropyl)triethoxysilane equal to 0.055 mmol/g, it can be concluded that the greater part of grafted amino groups interacted with 3-(Boc-amino)octanoic acid, and the spacers anchored on the Aerosil surface are mainly those shown in Figure 6.

**Figure 7:**
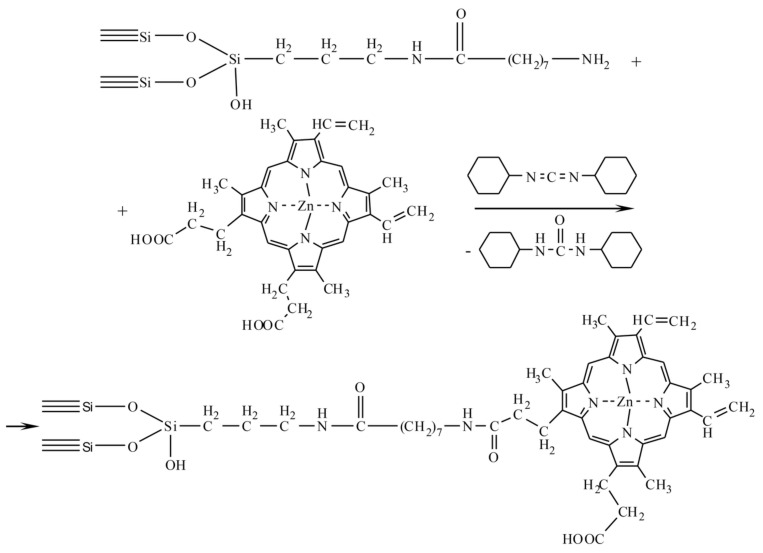
Immobilization of Zn-protoporphyrin.

**Figure 8:**
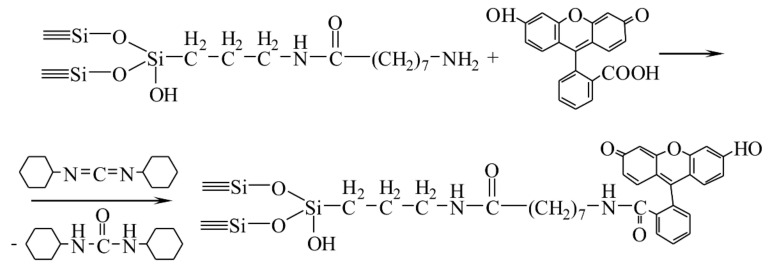
Immobilization of fluorescein.

Then, the obtained nanodispersed silica with grafted spacers is used to immobilize the anticancer drug Zn-protoporphyrin (Figure 7) and a fluorophore (fluorescein; Figure 8). These preparations are conjugated by using the carbodiimide method.

The sustained-release carriers are prepared by using matrices with various functional groups that ensure the covalent, ionic, and adsorption binding of biologically active substances [3].

The centers for immobilization of drugs that ensure covalent binding are formed by glutaraldehyde conjugated to the amino group of Aerosil. To ensure immobilization based on ionic bond formation, the aminated Aerosil is treated with succinic anhydride, and carboxyl groups are obtained on the carrier surface. Sorption immobilization is carried out on the surface of Aerosil A-380 containing silanol groups. Adenosine, which is widely used as a cardioprotector, was taken as a model drug [3].

**Figure 9:**
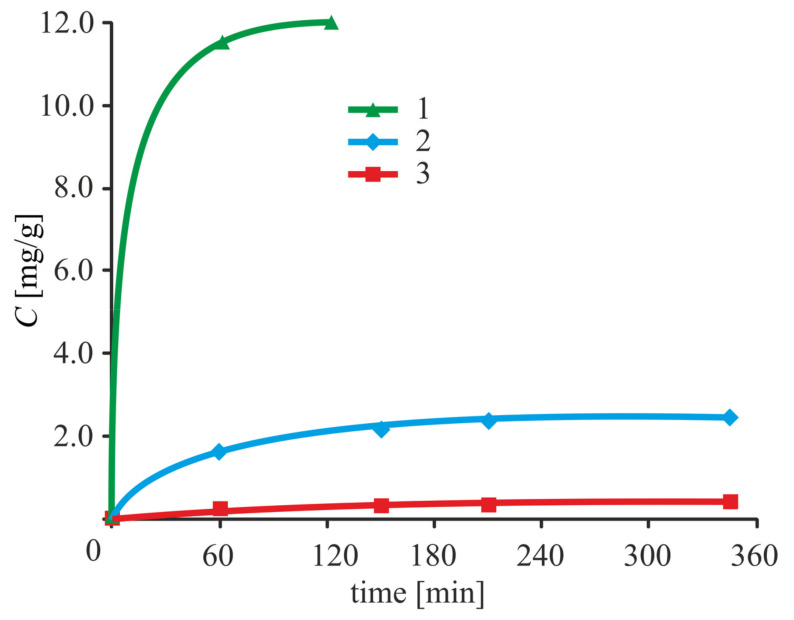
Dependence of the amount of adenosine (*C*) transferred to the solution from the surface of the nanoparticles, referring to a unit of weight in the desorption time for various immobilization methods: 1, adsorption; 2, covalent; and 3, ion binding [59].

The drug release kinetics (Figure 9) was studied by desorption of immobilized adenosine in Krebs–Henseleit buffer, with salt content close to that of the blood composition [59]. The most rapid release of the drug has been observed under adsorption immobilization, while the slowest was observed under ion binding. This fact can be explained by the instability of azomethine groups formed under adenosine binding by glutaraldehyde.

The immobilization of bradykinin as a cardioprotector (a peptide hormone) on aminated aerosil has been carried out by the glutaraldehyde method.

**Figure 10:**
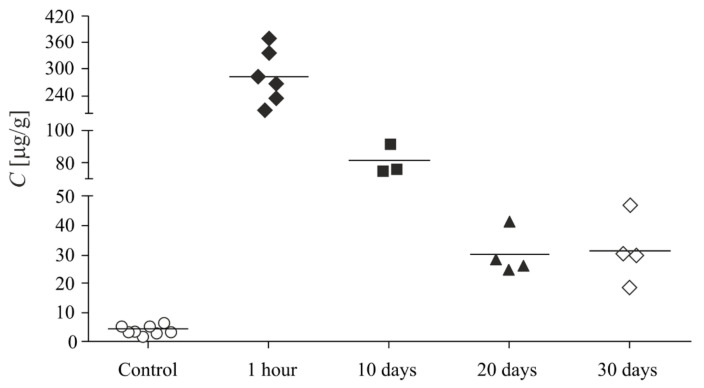
Silicon content (*C*) in the liver of animals at different times after the introduction of silica nanoparticles [60]: ○, Control; ◆, 1 h; ■, 10 days; ⯅, 20 days; and ◇, 30 days.

The obtained samples with immobilized biologically active substances (bradykinin, adenosine, fluorescein, and cardiogrin) were tested for toxicity, biocompatibility, and biodegradability of silica nanoparticles. It was shown that intravenous administration of nanodispersed particles to rats does not cause a significant change in hemodynamic parameters, such as blood pressure and heart rate, which indirectly indicates good tolerability of these drugs. Silicon content measurement by atomic absorption spectroscopy showed that, in 30 days, about 90% of the administrated silica was excreted by rats as a result of biodegradation (Figure 10) [60]. It was also found that adenosine (ADN) adsorption on silica nanoparticles increases the infarct-limiting effect of the drug (Figure 1a).

**Figure 11:**
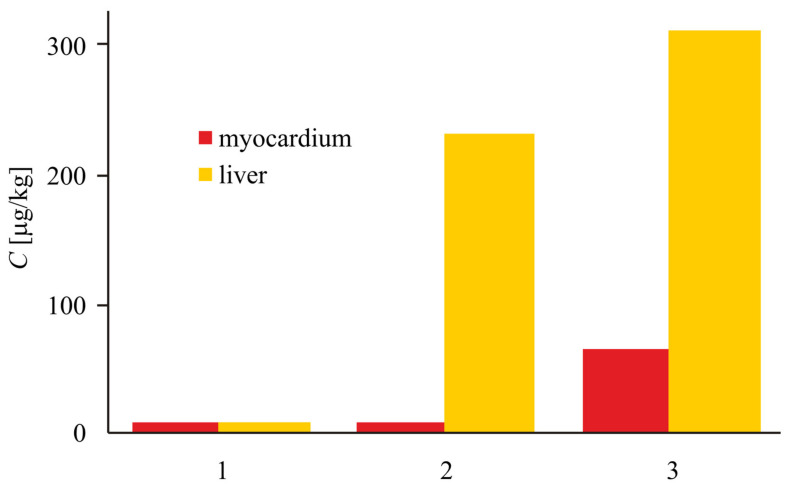
Silicon content (*C*) in the myocardium and liver of experimental animals of different groups, according to atomic absorption spectroscopy: 1, background levels; 2, sham operated animals, which underwent administration of silica nanoparticles; and 3, ischemia–reperfusion + silica nanoparticles (SNP) [12].

At the same time, a study on the distribution of silica in healthy rats and during myocardial ischemia has shown a significant increase in silica content in the damaged organ, which allows for the targeted delivery of drugs to the ischemic myocardium, based on modified aerosil nanoparticles (Figure 11) [11,12].

**Figure 12:**
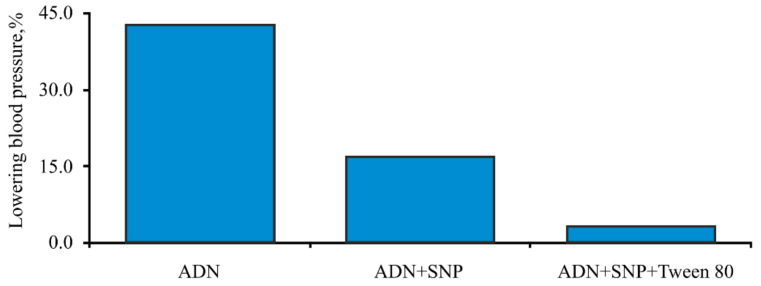
Hemodynamic effects of adenosine (ADN).

**Figure 13:**
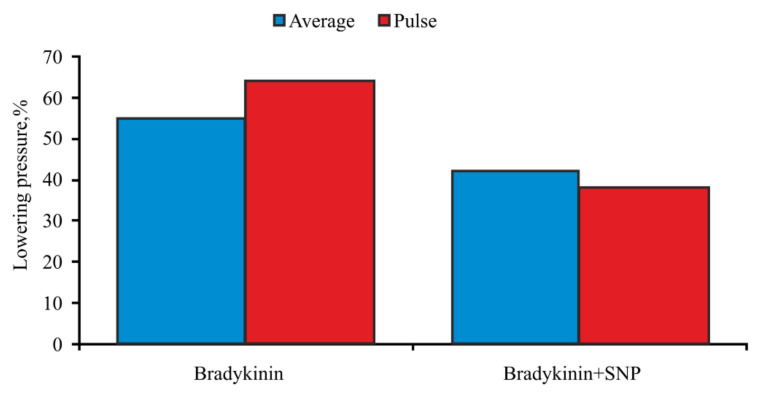
Hemodynamic effects of bradykinin.

It was found that the adsorption of adenosine (Figure 12) and bradykinin (Figure 13) on silica nanoparticles surface resulted in significant attenuation of the hypotensive effect.

The results of the analyses demonstrated the potential of using nanodispersed silica matrices as targeted drug delivery carriers.

Considering the high cost of peptide hormones—in particular, that of bradykinin—we investigated the possibility of obtaining silica-based matrices for peptide synthesis, guided by the idea that, in contrast to the known silica matrices, the obtained matrices can ensure separation of the target product under mild conditions, preventing the possible cleavage of peptide bonds and matrix destruction.

**Figure 14:**
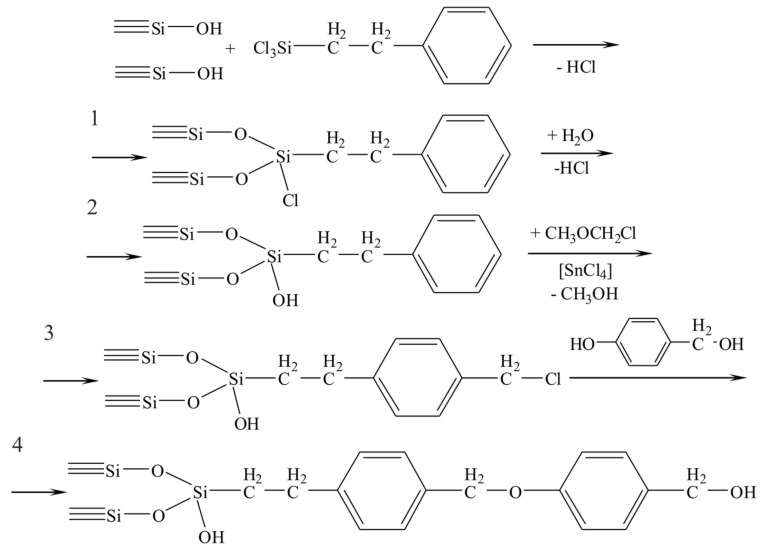
Scheme for the synthesis of the matrix to obtain peptides: 1, chemisorption of (2-Phenylethyl)trichlorosilane; 2, hydrolysis of chrosilyl groups; 3, chloromethylation of the aromatic ring; and 4, conjugation of p-hydroxybenzyl alcohol.

The developed synthesis technique includes a multistage process of chemical assembly on the initial silica surface (Figure 14), for which the silica gels KSK-2 and silochrome C-120 have been used [57]. The silica surface was extremely hydroxylated to achieve maximum functionalization at a later stage, through chemisorption of (2-Phenylethyl)trichlorosilane (Figure 14, scheme 1). The next step was the hydrolysis of chrosilyl groups (Figure 14, scheme 2), followed by chloromethylation of the aromatic ring, using chloromethyl methyl ether in the presence of SnCl_4_ (Figure 14, scheme 3). The last stage of the matrix synthesis was conjugation of p-hydroxybenzyl alcohol (Figure 14, scheme 4), which interacts with chloromethyl groups. It was found that about 50% of all chloromethyl groups entered into the reaction.

The silica matrices thus formed were tested in the synthesis of the glycylglycine dipeptide. It should be noted that glycine-based spacers have been widely used to immobilize biologically active compounds.

The dipeptide was prepared according to a classical solid-phase synthesis method by using Fmoc-glycine pentafluorophenyl ester. The first amino acid was conjugated by using the activated ester method. Then, the acid was released with morpholine-dimethylformamide solution, and the second amino acid was conjugated by using the same method. The dipeptide thus synthesized was separated from the carrier by reaction with a mildly acidic reagent (trifluoroacetic acid).

Consequently, using a chemical assembly method, new silica matrices have been synthesized for the preparation, immobilization, and targeted delivery of biologically active substances. These carriers can readily be used as platforms for developing preparations intended for theranostics, as they allow for the immobilization of marker compounds necessary to visualize lesions and to make diagnoses, as well as to achieve covalent and non-covalent immobilization of cardioprotectors and anticancer drugs. It is also worth noting that the developed synthetic approaches can be used to bind targeting ligands which ensure active targeted drug delivery; for example, the delivery of an increased concentration of silica nanoparticles containing cardioprotectors in the damaged parts of heart muscle can be achieved by immobilizing antibodies to annexin V, which is a highly specific marker of ischemic damage and is expressed on the ischemic focus surface [61].

## 5. Modern Platforms for Theranostics Applied for Treating Various Pathologies

There has recently been a significant upsurge in the design of platforms for theranostics, most of which are used for cancer therapy. Additionally, related studies have been devoted to cardiology, diabetes, treatment of the liver, kidneys, autoimmune, inflammatory, and neurological diseases. At the same time, to date, problems remain at the level of scientific research, due to the complexity of the chemical methods used to synthesize such systems. Here, we provide an overview of the most promising developments.

One study has proposed a new theranostic technique which includes the targeted delivery of relaxin (RLX) to the liver [13]. As is well-known, RLX has potent anti-fibrotic properties, but, at the same time, it has a suboptimal pharmacokinetic profile and serious side effects. In the above research, RLX was conjugated to PEGylated superparamagnetic iron oxide nanoparticles (RLX–SPION), and the specific binding/absorption of such nanoparticles by hepatic stellate cells (HSCs) was investigated, and the therapeutic effect of RLX–SPION on human HSCs in vitro and in vivo in a CCl4 model of induced liver cirrhosis in mice was evaluated. In a cell culture, RLX–SPION were bound to the surface of TGFβ-activated HSCs, after which the TGFβ-induced HSCs were internalized and inhibited differentiation, migration, and contraction. In vivo RLX–SPION significantly attenuated cirrhosis and showed increased contrast in MRI. In general, the research presented RLX–SPIONs as a novel theranostic platform which provides new opportunities for the diagnosis and treatment of cirrhosis.

An anticancer theranostics nanoplatform based on controlled near-infrared radiation (NIR) has been developed by encapsulating up-conversion nanoparticles (UCNPs) and the luminogen 2-(2,6-bis((E)-4-(phenyl(40-(1,2,2-triphenylvinyl)-[1,10-biphenyl]-4-yl)amino)styryl)-4H-pyran-4-ylidene)malononitrile (TTD) with an amphiphilic polymer having the characteristics of aggregation-induced emission (AIEgen) [14]. The cyclic peptide of arginine–glycine–aspartic acid (cRGD) was conjugated to obtain UCNP@TTD-cRGD nanoparticles. As an outcome of the work, the bioimaging and antitumor ability of UCNP@TTD-cRGD nanoparticles were assessed when illuminated with near-infrared radiation in an in vitro three-dimensional (3D) cancer spheroid mouse tumor model. With a close match between UCNP radiation and AIEgen absorption, the synthesized nanoparticles efficiently generated reactive oxygen species (ROS), even when excited through thick tissues. The developed NIR-regulated UCNP@TTD-cRGD can provide selective visualization of cancer cells and significantly inhibit tumor growth during NIR-regulated phototherapy, as compared with white-light excitation.

A theranostics nanosystem developed for highly selective therapy against tumors and for in situ tracking of fluorescence during cancer chemotherapy has been described [15]. The developed theranostic agent (RA-S-S-Cy) includes a disulfide bond as a cleavable linker, a near-infrared (NIR) active fluorophore acting as a fluorescent tracker, and the natural RA-V cyclopeptide acting as an active anticancer agent. Upon reaction with a high level of intracellular glutathione (GSH), a disulfide bridge is cleaved, resulting in a concomitant active release of the RA-V drug and a significant increase in NIR fluorescence. To further improve the RA-S-S-Cy tumor targeting and to increase the generation of reactive oxygen species, the RA-S-S-Cy, together with an oxygen-generating agent catalase, were included in the shell of the PLGA lactic acid copolymer targeted by peptide with (RGDfK), in order to obtain RA-S-S-Cy@PLGA nanoparticles.

An attempt has been made to combine the advantages of albumin nanoparticles and quantum dots (QD) to improve drug accumulation in tumors and the ability to perform strong fluorescence imaging on a single carrier [16]. Researchers have considered the problem of premature drug release from protein nanoparticles and the high toxicity of QD caused by the leakage of heavy metals. As a result, a cancer theranostics platform has been developed by combining a biocompatible albumin backbone with CdTeQD and mannose fragments, in order to enhance accumulation in tumors and reduce QD toxicity. The chemotherapeutic water-soluble drug pemetrexed (PMT) was conjugated, through a tumor-cleavable bond, to the albumin backbone for specific release into the tumor. In combination with the herbal hydrophobic drug resveratrol (RSV), a phospholipid complex was preliminarily formed, which ensured its physical encapsulation in albumin nanoparticles. The albumin–QD conjugate showed increased cytotoxicity and internalization in breast cancer cells, which can be traced due to their high quantum fluorescence yield and excellent visualization ability.

A new theranostic hybrid nanocomposite has been described [17], which includes an iron oxide core and a mesoporous silica shell (IO@MS) with an average size of 30 nm. The nanocomposite is coated with a layer of human serum albumin (HSA), and can be used for magnetic resonance imaging (MRI) and drug delivery. The porous structure of the IO@MS nanoparticles was loaded with the antitumor drug DOX, with 34 wt.% drug loading. To capture the drug, a dense HSA coating bound by isobutyramide was applied. It has been shown that this protein nanoassembly is destroyed by proteases, thus releasing DOX. The effect has been proven in a three-dimensional cell model, using confocal imaging. Cytotoxicity was observed in studies of spheroid growth inhibition in liver cancer cells.

Another study has focused on a metal organic framework (MOF) combined with hollow mesoporous organosilica nanoparticles (HMONs), using an intermediate layer of polydopamine (PDA) to form molecular organic/inorganic hybrid nanocomposites (HMONs–PMOF) [18]. Doxorubicin hydrochloride DOX and indocyanine green (ICG) were separately loaded into the HMON internal cavity and on the MOF outer porous shell. The resulting double drug-loaded nanocomposites (DOX/ICG@HMONs–PMOF) have shown good photothermal properties and pH/NIR-initiated DOX release. In addition, in vitro cell experiments have confirmed that HMONs–PMOFs can efficiently deliver DOX to cancer cells. When released into cancer cells, the photothermal effect of DOX/ICG@HMONs–PMOF can cause lysosomal rupture, thereby facilitating the “exit from lysosomes” process and accelerating DOX diffusion in cytoplasm. To obtain effective DI@HMONs–PMOF accumulation, the tumor location was investigated, in terms of the benefits of using iron ions coordinated on PDA, and ICG enclosed in MOF, magnetic resonance (MR), and photoacoustic (PA) dual-mode imaging. The results also indicated that ICG attached to nanoparticles can improve the capabilities of MR imaging with the prepared nanocomposites.

Another research has shown that the use of glucocorticoids (GC), as a component of nanoparticles, can improve delivery to inflamed areas, thus increasing their effectiveness and minimizing the required dose, consequently reducing the associated side-effects [19]. The nanoparticles proposed in the research consist of GC betamethasone phosphate (BMP) and fluorescent dye DY-647 (BMP-IOH-NP). These nanoparticles have been recommended for the more effective treatment of inflammation while monitoring the in vivo delivery. The uptake of BMP-IOH-NP by macrophages was analyzed by using fluorescence and electron microscopy. Lipopolysaccharide-stimulated cells were treated for 48 h with BMP-IOH-NP (1 × 10^−5^—1 × 10^−9^ M), BMP, or dexamethasone (Dexa). The drug efficiency was evaluated by measuring the level of interleukin 6l. Mice with zymosan-A-induced limb inflammation were injected intraperitoneally with BMP-IOH-NP (10 mg/kg), and mice with ovalbumin-induced allergic airway inflammation (AAI) were treated intranasally with BMP-IOH-NP, BMP, or Dexa (2.5 mg/kg each). Efficacy assessment was performed in vivo by limb volume measurement and ex vivo by measuring the limb mass in mice injected with zymosan-A, or in the AAI model by in vivo evaluation of lung function by radiography and cell count in bronchoalveolar lavage fluid. BMP-IOH-NP delivery to the lungs of AAI mice was monitored by in vivo optical imaging and fluorescence microscopy. It was shown that the synthesized BMP-IOH-NP nanoparticles can be successfully used in anti-inflammatory theranostics.

FHMP nanoparticles (FHMP NPs) have been synthesized for sonodynamic therapy (SDT) and PA imaging of tumors by integrating melanin nanoparticles (MNPs, a component for PA imaging) into the shell of hematoporphyrin monomethyl ether (HMME, a component for improving SDT) [20]. Then, the nanoparticles were protected with poly(lactic-co-glycolic acid) (PLGA) and additionally functionalized with folic acid (FA)—a tumor-oriented ligand.

The synthesized FHMP NPs with wide optical absorption not only possess high ability to enhance contrast during PA imaging, but also demonstrate significant SDT efficiency. The PLGA-based nanoplatform improved the HMME light stability and sonodynamic characteristics, as well as facilitating MNP delivery to the tumor area. The sonosensitizer, which is assisted by ultrasound irradiation, generates ROS-mediated cytotoxicity against tumor cells. It has been demonstrated that, at the cellular level in in vitro and in vivo tumor xenograft mouse models with tumors, FHMP NPs contributed to the selective ROS killing effect in tumor cells and played an active role in the suppression of tumor growth.

Hydrophobic superparamagnetic iron oxide (SPIO) nanoparticles have been obtained by using the thermal decomposition method [21]. They were coated with 1,2-distearoyl-sn-glycero-3-phosphoethanolamine-N-[methoxy(polyethyleneglycol)-2000] (DSPE-PEG 2000) and DOX, using a thin-film hydration technique, followed by ICG loading into phospholipid layers. In vitro biocompatibility and antitumor efficacy were assessed by using MTT analysis. In vivo fluorescence and magnetic resonance imaging (MR) were used to assess penetration across the blood–brain barrier (BBB) and accumulation in brain tumor tissue.

The obtained multifunctional nanoparticles had an average diameter of 22.9 nm, zeta potential of –38.19 mV, and were able to provide a sustained release of DOX. In vitro experiments showed that SPIO@DSPE-PEG/DOX/ICG nanoparticles effectively increased cellular uptake of DOX, as compared to free DOX. In vivo fluorescence and MRI showed that the nanoparticles not only effectively overcame the BBB, but also selectively accumulated at the tumor location.

A multifunctional theranostics nanoplatform has been developed on the basis of gold nanoparticles (NF) stabilized by poly(amidoamine) dendrimer of the fifth branching order (G5), to which ultra-fine iron oxide nanoparticles (USIO) were added [22]. This composition can be used for combined photothermal therapy (PTT) and radiation therapy (RT) under the control of multimodal imaging, namely T1-weighted magnetic resonance (MR)/computed tomography (CT)/PA imaging. Gold nanoparticles stabilized by the G5 dendrimer and citric acid–stabilized USIO were obtained separately. Then, they were mixed at a certain molar ratio (Fe:Au) with the formation of complexes. The complexes were exposed to the solution for the growth of gold nanoparticles. The remaining dendrimer terminal amine groups were then acetylated. The resulting DSNF-stabilized Fe_3_O_4_/Au had an average diameter of 99.8 nm, showed good colloidal stability, cytocompatibility, and near-infrared absorption. The unique structure and composition of DSNF Fe_3_O_4_/Au provided high relaxivity r_1_ (3.22 mM^−1^ s^−1^) and a photothermal conversion efficiency (82.7%), which allows them to be used as a theranostics nanoplatform for multimodal MR/CT/PA imaging, PTT, and radiotherapy (RT) of tumors with improved therapeutic efficiency.

Red fluorescence ZnO nanoparticles have been synthesized by using the polyol method in boiling trimethylene glycol (TREG) with zinc acetate [23]. ZnO nanoparticles were grafted with a polyglycidol layer by ring-opening polymerization of glycidol (ZnO-PG). As calculated from the thermogravimetric analysis data, the weight ratio of the grafted PG was about 68 wt.%. Then, ZnO-PG was conjugated to the arginine–glycine–aspartate (RGD) peptide through stepwise organic reactions. The anticancer drug DOX was immobilized on ZnO-PG-RGD to form ZnO-PG-RGD/DOX, with particle size of 21.8 ± 0.9 nm. The drug-release rate reached 70.6% within 48 h, at a pH of 5.2, which was more than three times the value at a pH of 7.4. The grafted PG layer not only significantly improved the dispersibility, but also inhibited the uptake of ZnO nanoparticles by U87MG and HeLa cells. In contrast, ZnO-PG-RGD was selectively absorbed by U87MG, rather than by HeLa cells, demonstrating the obvious targeting.

In another experiment, a porphyrin grafted lipid (PGL) ring has been used to load DOX and to apply synergistic chemo-PDT [24]. Self-assembled liposomal PGL nanoparticles with hydrophilic cores were used to encapsulate DOX, using a pH gradient. The encapsulation efficiency was ~99%. The resulting PGL–DOX nanoparticles were highly stable and were successfully removed from the endolysosomal compartment after laser irradiation to release DOX in the cytosol. The PGL–DOX nanoparticles had good cellular uptake, a chemo-photodynamic response, and the ability to visualize fluorescence in various cell lines. After exposure to laser radiation, a significant decrease in viability of cells treated with a low molar concentration of PGL–DOX NPs was observed. In addition, in vivo experiments performed on a tumor xenograft model in mice demonstrated the ability of PGL–DOX accumulation in tumors due to passive targeted delivery. Through fluorescence imaging, the PGL–DOX biodistribution in tumors and in major body organs were also easily monitored in real-time in vivo.

In Reference [27], a theranostic platform (MnO_2_-SiO_2_-APTES and Ce6; MSA & C) based on MnO_2_ nanoflowers was synthesized, which provides synergistic therapy guided by MRI, including PDT and PTT in the second near-infrared window (NIR-II). Nanoflowers refer to chemical compounds that form structures that, under TEM, resemble flowers (or, in some cases, trees), sometimes called nanobouquets or nanotrees. In the study, MnO_2_ nanoflowers have been proposed for the first time as a photothermal NIR-II agent. In the MSA & C system, MnO_2_ nanoflowers were used to efficiently load the photosensitizer, relieve tumor hypoxia, and conduct NIR-II PTT tumor imaging. The large amount of photosensitizers, as well as reduced tumor hypoxia and hyperthermia, contributed to the improvement of PDT. Positively charged APTES was used to stimulate cellular uptake, further enhancing the treatment efficacy.

Another study focused on the combination of hydrophobic and electrostatic non-covalent interactions for bimodal fluorescence/photoacoustic imaging of breast cancer [28]. The authors integrated multicomponent hyaluronic acid (HA), protamine (PS), nanodiamonds (ND), curcumin (Cur), and ICG into a single nanoplatform (designated as HPNDIC). To achieve this goal, a two-stage build strategy was used. At the first stage, PS was used to modify ND clusters with the formation of positively charged PS @ ND (PND), with the simultaneous encapsulation of natural low molecular weight drug Cur and photosensitive ICG. Second, HA was adsorbed onto the outer surface of the PNDIC through charge complexation, in order to provide tumor-targeting ability. The resulting HPNDIC had uniform size, high drug-loading capacity, and excellent colloidal stability. It has been found that, under near-infrared irradiation conditions, ICG can be used for both PTT and PDT, resulting in an increased efficacy of Cur therapy (both in vitro and in vivo) with good biocompatibility. The presence of ICG and the accumulation of HPNDIC in vivo can be used for imaging by bimodal fluorescence/photoacoustic imaging.

It has been shown [30] that multifunctional theranostic nanostructures, consisting of superparamagnetic iron oxide and gold nanoparticles scuffed inside graphene oxide nanoflasts, can be used for double photo/radiation therapy due to near-infrared absorption of graphene oxide for photothermal therapy and radiosensitization by gold nanoparticles for enhanced radiation therapy. At the same time, this nanoplatform can also be detected by MRI imaging, due to the presence of iron compound nanoparticles. In a mouse carcinoma model, the platform showed 1.85 and 1.44 times higher therapeutic efficacy in combined photo and radiation therapy, respectively, compared to pure graphene oxide, which led to the complete destruction of tumors.

A study has investigated the possibility of developing chitosan nanococktails containing nanoparticles of both nanocerium and superparamagnetic iron oxide [31]. Nanocerium, which is capable of trapping reactive oxygen species, and iron oxide nanoparticles, used as imaging agents for MRI, were synthesized separately. Theranostics platforms have been constructed through two different mechanisms: electrostatic self-assembly and ionic gelation. These theranostic nanococktails have demonstrated the efficient uptake of reactive oxygen species and MRI contrast as a potential platform for the treatment and diagnosis of various diseases.

As a platform for theranostics, dendrimer-modified gold nanorods for combined gene therapy and photothermal therapy for colon cancer have been synthesized [32]. Gold nanorods grafted with polyamidoamine dendrimers (PAMAM, G3) have been modified with the GX1 peptide (cyclic 7-mer peptide, CGNSNPKSC). The resulting nanoplatform has been proposed as a gene-to-gene vector (FAM172A, which regulates the proliferation and apoptosis of colon cancer cells) for combined photothermal and gene therapy for colon cancer cells (i.e., HCT-8 cells). In addition, the computed tomography function using this platform can provide diagnostic data for colon cancer.

Porphyrin lipids have been used [33] to create several multimodal nanoparticle platforms, including liposome-like porphysomes (water core), porphyrin nanodrops (liquefied gas core), and ultra-small porphyrin lipoproteins. Porphyrin lipids were used to stabilize the water/oil interface to create porphyrin–lipid nanoemulsions with paclitaxel (PTX) loaded into an oil core (PLNE–PTX). This can facilitate combined PDT and chemotherapy at the same time.

PTX (3.1 wt.%) and porphyrin (18.3 wt.%) were efficiently loaded into PLNE–PTX, forming spherical core–shell nanoemulsions that were 120 nm in diameter. PLNE–PTX showed stability upon delivery, resulting in high tumor accumulation (~5.4 ID%/g) in KB tumor mice. The PLNE–PTX combination therapy better inhibited tumor growth (78%) in a selective manner, compared to PDT (44%) or chemotherapy (46%), 16 days after treatment. In addition, the fourfold reduced dose of PTX (1.8 mg PTX/g) in the PLNE–PTX combination therapy platform has shown increased therapeutic efficacy, compared to taxol 7.2 mg PTX/kg, which may reduce the associated side effects. PLNE–PTX fluorescence allows for real-time tracking of the penetration of nanoparticles into the tumor.

Researchers have developed metal nanoparticles in combination with cyclodextrin as a new platform to reduce the effects of traditional chemotherapy, such as stomach irritation, hair loss, neurotoxicity, and so on [35]. Encapsulating drugs with metal nanoparticles can help to overcome the limitations of chemotherapy and efficiently transport anticancer drugs to the target site. This is due to various advantages, such as optimal size, surface morphology, higher conductivity, and in vivo stability. Such platforms allow for controlled drug release under the influence of NIR radiation or a magnetic field. Some commonly used chemotherapeutic agents, such as doxorubicin, paclitaxel, methotrexate, and so on, are rapidly degraded due to their hydrophobic nature and are unstable in vivo. Cyclodextrin provides structural compatibility for the encapsulation of such hydrophobic drugs and improves their loading capacity, solubility, and stability without exhibiting any systemic toxicity.

Two-dimensional intermetallic PtBi/Pt nanoplates (PtBi NP) have been developed as a therapeutic platform for in situ oxygen production, thus overcoming tumor hypoxia to enhance PTT/RT [36]. As they possess a high X-ray attenuation coefficient, PtBi NPs have demonstrated high sensitization characteristics at RT. PEGylated PtBi NPs (PtBi-PEG) exhibit high biocompatibility, increased circulation time in the blood, and increased accumulation in the tumor. PtBi-PEGs have also been used for tri-modal NIR contrast enhancement, PA, and X-ray imaging.

## 6. Conclusions

The concept of theranostics was introduced by Funkhouser in 2002, defined as the integration of two modalities—that is, therapy and medical imaging—into a single “package” of a material intended to overcome undesirable variations in biodistribution and therapeutic efficacy [62]. Theranostic materials provide a window for monitoring the pharmacokinetics and pharmacodynamics of the drug introduced into the body. This concept was initially largely focused on cancer therapy, but it was later expanded to other pathologies.

**Figure 15:**
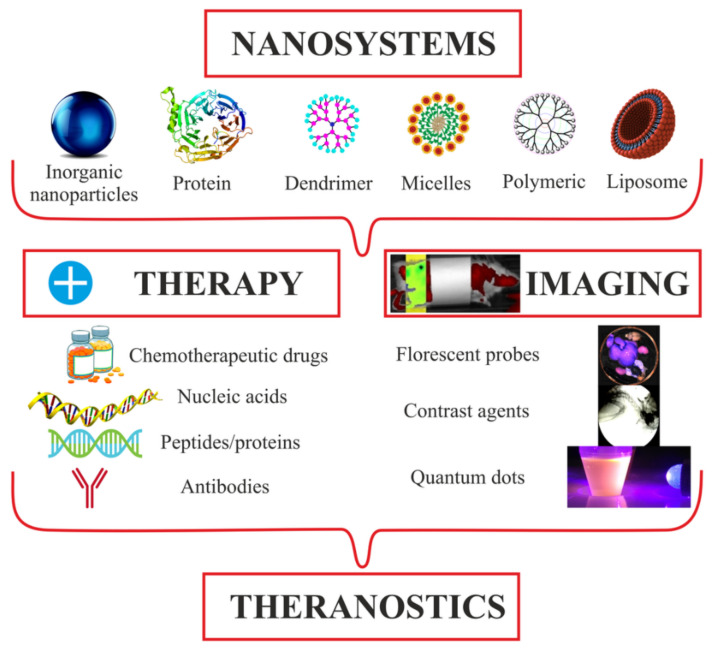
Generalized scheme of theranostics in oncology.

For theranostics applied to oncological diseases, the methods for synthesizing theranostic constructs have been systematized [63]. This scheme is suitable for all theranostic constructs, if, by treatment, we mean drugs in each specific area (Figure 15).

In the ever-growing field of personalized medicine, nanotechnology plays a vital role by integrating diagnostic and therapeutic functions into a single system called nanotheranostics [64]. Using theranostic nanomaterials, it is possible to achieve the correct diagnosis and to develop an appropriate therapeutic intervention, while simultaneously targeting the diseased cells during systemic circulation, evading the immune system, and visualizing pathological areas [65].

**Figure 16:**
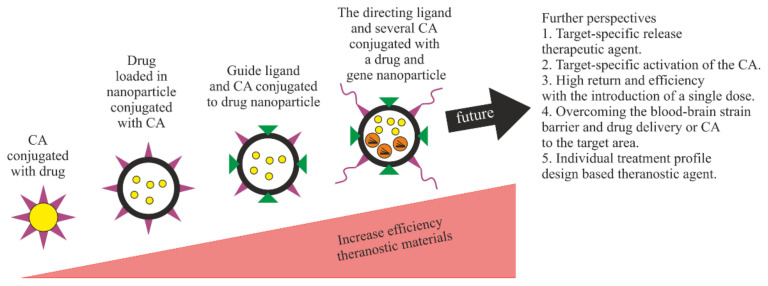
Schematic illustration of advances in the field of nanotheranostics. CA, contrast agent.

Figure 16 shows that nanosystems can be used for the individualized treatment of a particular patient and for increasing survival. The theranostic efficiency of nanomaterials gradually increases along with the use of “smart” and novel biomaterials [62]. Theranostic constructs can be produced from materials that respond to biological environment, such as temperature, pH, an enzyme, or a specific target group that provides systemic release of the drug and reduces toxicity in healthy tissues. All of these are questions concerning the future, such as the transfer into clinical practice.

At present, a huge number of theranostic constructs have been invented in such areas as cancer therapy, inflammatory diseases, autoimmune disorders, diabetes, cardiovascular diseases, neurological disorders, and liver and kidney therapy. The introduction of these systems into clinical practice is tempered by the associated complexity of synthesis, which means that the time has not yet come for industrial production.

## Figures and Tables

**Table 1:** Nanomaterials used for theranostics systems.

Material	Application Area
Silica nanoparticles containing cardioprotectors	Cardiovascular diseases [11,12]
PEGylated superparamagnetic iron oxide nanoparticles	Liver cirrhosis [13]
Amphiphilic polymer with luminogen, cyclic peptide of arginine–glycine–aspartic acid	Cancer [14]
PLGA lactic acid copolymer, natural RA-V cyclopeptide, fluorescent tracker, oxygen-generating agent catalase	Cancer [15]
Albumin nanoparticles and quantum dots, mannose fragments, pemetrexed	Cancer [16]
Iron oxide core with mesoporous silica shell, layer of human serum albumin, doxorubicin	Cancer [17]
Metal organic framework (MOF) combined with hollow mesoporous organosilica nanoparticles, indocyanine green, doxorubicin	Cancer [18]
Nanoparticles consist of GC betamethasone phosphate (BMP) and fluorescent dye DY-647	Anti-inflammatory theranostics [19]
Melanin nanoparticles with the shell of hematoporphyrin monomethyl ether, poly(lactic-co-glycolic acid), additionally functionalized with folic acid	Sonodynamic therapy [20]
Superparamagnetic iron oxide nanoparticles with polyethylene glycol, doxorubicin (DOX), indocyanine green, phospholipid layers	Brain tumor [21]
Gold nanoparticles stabilized by poly(amidoamine) dendrimer with ultrafine iron oxide nanoparticles	Combined photothermal therapy and radiation therapy [22]
ZnO nanoparticles with polyglycidol layer, (arginine–glycine–aspartate) peptide, DOX	Cancer [23]
Porphyrin grafted lipid (PGL) ring with DOX	Synergistic chemo-photodynamic therapy (PDT) [24]
Dendrimer architectonics	Cancer and neurodegenerative diseases [25]
Gadolinium	Cancer, MRI [26]
MnO_2_ nanoflowers	Photothermal/photodynamic therapy [27]
Multicomponent-assembled nanodiamond hybrids	Cancer [28]
Polymer hydrogels	Cancer [29]
Graphene oxide nanoflakes	Cancer, MRI [30]
Chitosan nanococktails containing both ceria and superparamagnetic iron oxide nanoparticles	Reactive oxygen species-related theranostics [31]
Dendrimer-modified gold nanorods	Combinational gene therapy and photothermal therapy of tumors [32]
Porphyrin-lipid stabilized paclitaxel nanoemulsion	Chemotherapy [33]
Hyaluronic acid–based nanoplatforms for DOX	Cancer [34]
Cyclodextrins-modified metallic nanoparticles	Cancer [35]
Two-dimensional intermetallic PtBi/Pt core/shell nanoplates	Cancer [36]
Janus metallic mesoporous silica nanoparticles	Cancer [37]

## Data Availability

Not applicable.
